# Long noncoding RNA Gpr137b-ps mediates 15-PGDH-induced angiogenesis in brain microvasculature following tMCAO in mice

**DOI:** 10.1371/journal.pone.0349591

**Published:** 2026-07-24

**Authors:** Di Wang, Guojian Zhang, Haiyan Gou, Yawei Wang, Wenye Liang, Yangyue Cao, Yujuan Jiao

**Affiliations:** 1 Department of Neurology, The First Hospital of Tsinghua University, School of Clinical Medicine, Tsinghua Medicine, Tsinghua University, Beijing, China; 2 Department of Neurology, The Second Affiliated Hospital of Harbin Medical University, Harbin, Heilongjiang, China; 3 Department of Neurology, Beijing Tongren Hospital, Capital Medical University, Beijing, China; Helwan University, EGYPT

## Abstract

**Background:**

Angiogenesis is essential for recovery following ischemic stroke, as brain microvascular endothelial cells (BMVECs) drive vascular repair. 15-hydroxyprostaglandin dehydrogenase (15-PGDH) is a major regulator of endothelial function in some vascular contexts, but it is unknown whether 15-PGDH plays a significant role in angiogenesis following stroke. Notably, based on preliminary bioinformatics prediction of their 3’UTR interaction, we hypothesize that Gpr137b-ps—a pseudogene-derived lncRNA originating from Gpr137b—forms a regulatory link with 15-PGDH to modulate this process (i.e., post-stroke angiogenesis) by regulating 15-PGDH expression, though its specific functional mechanism in this context remains largely unknown.

**Methods:**

To address this gap, we used mouse middle cerebral artery occlusion (MCAO) model in vivo and BMVEC oxygen-glucose deprivation/reoxygenation (OGD/R) model in vitro. We modulated 15-PGDH via siRNA and Gpr137b-ps via mimics, and assessed angiogenesis using migration, tube formation, and cell-cycle assays, with expression and localization of key molecules verified by standard molecular techniques.

**Results:**

Both MCAO and OGD/R successfully induced ischemia, with significant upregulation of 15-PGDH and Gpr137b-ps in ischemic tissues and BMVECs (P < 0.05). Despite this concurrent upregulation, 15-PGDH promoted angiogenesis (silencing reduced BMVEC proliferation, migration, and tube formation; P < 0.01), whereas Gpr137b-ps overexpression suppressed 15-PGDH protein levels and inhibited angiogenesis—effects that were rescued by re-expressing 15-PGDH.

**Conclusion:**

Together, our findings identify Gpr137b-ps/15-PGDH as a key feedback regulatory axis: while both Gpr137b-ps and 15-PGDH are upregulated under ischemia, Gpr137b-ps (a negative regulator of angiogenesis) suppresses 15-PGDH (a pro-angiogenic factor) to repress excessive post-stroke angiogenesis via inhibiting BMVEC proliferation, migration, and cell-cycle progression. The data support an association and partial dependency between this axis and angiogenic processes, highlighting it as a potential therapeutic target (e.g., via inhibiting Gpr137b-ps) for vascular repair after ischemic stroke.

## 1 Introduction

Ischemic stroke, accounting for ~85% of all strokes, causes severe microvascular injury and is a leading cause of long-term disability. Notably, angiogenesis (formation of new blood vessels and collateral circulation) is a key endogenous repair mechanism driving post-stroke recovery by restoring blood supply to ischemic brain regions—this process relies on the proliferation, migration, and differentiation of brain microvascular endothelial cells (BMVECs) [1]. Despite its importance, the molecular mechanisms regulating brain microvascular endothelial angiogenesis after stroke remain poorly understood.

15-hydroxyprostaglandin dehydrogenase (15-PGDH), a NAD-dependent enzyme involved in the metabolism of arachidonic acid, catalyzes the oxidative degradation of bioactive prostaglandins (e.g., PGE_2_, PGF_2_α)—key mediators of endothelial proliferation, migration, and vascular permeability [2]. By regulating prostaglandin turnover, 15-PGDH modulates downstream signaling pathways (e.g., cAMP/PKA and PI3K/Akt) that govern endothelial cell behavior, and it has been implicated in hypoxia-induced angiogenesis [2]. We previously reported that ischemia-induced hypoxia led to increased expression of 15-PGDH in the brain, which corresponded to the contraction of cerebrovasculature [3]. Also, we provided evidence that 15-lipoxygenase (15-LO) involved in the arachidonic acid pathway either stimulated endothelial proliferation in brain vasculature or regulated other vascular-related processes [4]. Long noncoding RNAs (lncRNAs) and pseudogenes are increasingly recognized as important post-transcriptional regulators of proliferation, migration, and apoptosis, with pseudogenes functioning as key regulators in disease processes [5], and lncRNAs are shown to promote angiogenesis under oxygen-glucose deprivation/reoxygenation conditions [6]. Moreover, the pseudogene Gpr137b has been associated with vascular repair–related processes and regulation of autophagy in macrophages that contributed to atherosclerosis [7], but the role of its transcript, the pseudogene-derived lncRNA Gpr137b-ps, in post-stroke angiogenesis remains uncharacterized. As preliminary bioinformatics analysis via starBase showed [8] predicted an interaction between Gpr137b-ps and 15-PGDH’s 3’UTR, we hypothesize that Gpr137b-ps may post-transcriptionally regulate 15-PGDH expression, thereby modulating brain microvascular endothelial cell (BMVEC) function and post-stroke angiogenesis.

To address the knowledge gap regarding pseudogene-derived lncRNA regulation of 15-PGDH in post-stroke angiogenesis, this study aims to: (1) verify the interaction between Gpr137b-ps and 15-PGDH; (2) investigate whether Gpr137b-ps modulates 15-PGDH expression and function in BMVECs under ischemic/hypoxic conditions (in vitro OGD/R and in vivo MCAO models); (3) explore the role of the Gpr137b-ps/15-PGDH axis in regulating post-stroke angiogenesis via BMVEC proliferation, migration, and cell-cycle progression; and (4) evaluate the potential of this axis as a therapeutic target for vascular repair after ischemic stroke. The core hypothesis of this study is that lncRNA Gpr137b-ps directly binds to the 3’UTR of 15-PGDH to post-transcriptionally inhibit its expression, thereby suppressing angiogenesis in brain microvascular endothelial cells after ischemic stroke. The rationale for establishing in vitro OGD/R and in vivo MCAO models is to mimic ischemic/hypoxic stress of stroke and verify the expression and interaction of Gpr137b-ps and 15-PGDH under pathological conditions; the rationale for gain- and loss-of-function assays (mimic transfection, siRNA silencing) is to confirm the regulatory effect of Gpr137b-ps/15-PGDH axis on angiogenesis; the rationale for luciferase reporter assay is to validate the direct binding between Gpr137b-ps and 15-PGDH 3’UTR; the rationale for angiogenesis-related assays (tube formation, cell cycle, proliferation) is to evaluate the functional role of this axis in endothelial angiogenic behavior.

## 2 Materials and methods

### 2.1 The included materials

Bovine brain microvascular endothelial cells (BMVECs) were acquired from ScienCell Research Laboratories (Carlsbad, CA, USA, Cat# 1000) and cultured in RPMI 1640 medium with 15% fetal bovine serum (FBS). Factor VIII (FVIII) immunocytochemistry was performed to verify the endothelial identity of BMVECs and confirm culture purity, with a positive staining rate > 95%. Cells were used between passages 3 and 5 to maintain phenotypic stability, and mycoplasma testing was conducted every 2 weeks. A biological replicate was defined as an independent culture preparation, and “n” represents the number of independent cultures rather than multiple plates from the same culture session. Cells were used once they reached 80% confluence.

### 2.2 Antibodies and reagents

Six-week-old male C57BL/6 mice (Beijing Vital River Laboratory Animal Technology, Beijing, China, Cat# SCXK-2019–0010) were housed under standard conditions (12 h light/dark cycle; 21 ± 1 °C). Transient MCAO for 60 min with subsequent reperfusion was performed as previously assessed [8]. Mice were anesthetized with 4% isoflurane for induction and maintained with 1% isoflurane. Cerebral blood flow (CBF) was monitored with laser Doppler flowmetry (CBF < 70% or evidence of hemorrhage = exclusion criteria). Mice were sham-operated with thread insertion only, with no occlusion. Rectal temperature was maintained at 37 ± 0.5 °C.

All primary and secondary antibodies used in this study were validated for specificity, with details as follows: 1. Primary antibodies: Anti-15-PGDH (Abcam, ab211745, 1:200) – validated via Western blot (WB) using recombinant 15-PGDH protein and knockout cell lysates as negative control (per manufacturer’s protocol and [11]); Anti-CD31 (Abcam, ab9498, 1:100) – validated by immunofluorescence colocalization with endothelial cell-specific marker Factor VIII; Anti-Ki67 (Abcam, ab15580, 1:200) – validated via WB and IHC with proliferating vs. quiescent BMVECs; Anti-15-LO (Cayman Chemical, 32800, 1:100) – validated per manufacturer’s technical data sheet and published studies [10]; Anti-PCNA, Anti-VEGF, Anti-cyclin A/D, and Anti-β-actin (all from Cell Signaling Technology, Cat# 2586, 2932, 4656, 12202, 4970; dilution 1:1000 for WB) – validated using positive control lysates and β-actin as internal loading control. 2. Secondary antibodies: Alexa Fluor–conjugated (Invitrogen, Cat# A-11029, A-11036, 1:500) and HRP-conjugated (ZSGB-Bio, Cat# ZB-2301, 1:200) secondary antibodies – validated for no cross-reactivity with non-target species. 3. Experimental controls: Negative controls (omission of primary antibody) were included in all IHC/IF assays to rule out non-specific binding; β-actin served as internal control for WB to ensure equal loading; recombinant proteins or known positive samples were used to confirm antibody binding specificity.

### 2.3 Oxygen-glucose deprivation/reoxygenation (OGD/R) model

The BMVECs used in this study are bovine-derived (ScienCell Research Laboratories, Carlsbad, CA, USA, Cat# 1000). Factor VIII antibody staining was performed to confirm both the identity of the endothelial cell population and culture purity, with a positive staining rate > 95%. Cells were used between passages 3 and 5 to maintain their native phenotypic and functional characteristics, and mycoplasma testing was conducted every 2 weeks using a commercial detection kit (Thermo Fisher Scientific, Waltham, MA, USA, Cat# A10254), with all cell cultures verified as mycoplasma-negative throughout the study. For this research, a biological replicate is defined as an independent culture preparation, and “n” refers to the number of independent cultures rather than multiple plates derived from the same culture session. For the oxygen-glucose deprivation/reoxygenation (OGD/R) model (hereafter referred to as OGD/R), BMVECs were flushed twice with glucose-free HBSS (pH 7.4), then incubated in pre-gassed HBSS and placed in a tri-gas incubator (Thermo Fisher Scientific, Waltham, MA, USA) with precise control of gas composition (5% CO_2_, 95% N_2_, 0% O_2_) to maintain a stable hypoxic environment at 37 °C for 60 min; for the reoxygenation phase, cells were returned to normoxia with FBS-supplemented medium for another 24 h, while control cells were incubated with glucose-supplemented HBSS under normoxic conditions. The 60-minute OGD duration was selected based on our preliminary experiments and supported by published studies [9] showing that this duration effectively induces hypoxic stress in BMVECs, which is consistent with previous reports demonstrating that 30–60 minutes of OGD is sufficient to trigger angiogenic and cell-cycle responses in brain microvascular endothelial cells; in contrast, longer durations (≥4 hours) often lead to excessive cell death that masks the regulatory effects of the Gpr137b-ps/15-PGDH axis.

### 2.4 Middle cerebral artery occlusion (MCAO) model

Six-week-old male C57BL/6 mice were subjected to transient MCAO as previously described [9], with key parameters specified as follows: a 6−0 nylon monofilament suture (with a rounded tip) was inserted into the external carotid artery and advanced to occlude the middle cerebral artery for 60 min, followed by reperfusion for 72 h until sacrifice (the endpoint for all in vivo analyses). Anesthesia was induced with 4% isoflurane (mixed with 100% oxygen) and maintained at 1–2% isoflurane during surgery to ensure stable anesthesia and minimize intraoperative discomfort, while cerebral blood flow was monitored by laser Doppler flowmetry < 70% = inclusion criteria). Sham-operated mice underwent thread insertion without occlusion. A total of 60 male C57BL/6 mice were randomly divided into two main groups: sham-operated group (n = 30) and MCAO group (n = 30). For immunohistochemical analysis, 10 mice per group were used; for Western blot and flow cytometry analysis, 6 mice per group were allocated. The sample size was determined based on previous similar studies [9,11] and power analysis (G*Power 3.1) to detect a 30% difference in angiogenic outcomes (e.g., tube formation length) with α = 0.05 and power = 0.85, ensuring sufficient statistical power to identify meaningful group differences. Inclusion criteria: Mice with cerebral blood flow (CBF) < 70% after occlusion (confirmed by laser Doppler flowmetry) and survival for 72 hours post-surgery. Exclusion criteria: Mice with CBF ≥ 70% (insufficient occlusion), intraoperative hemorrhage, postoperative weight loss > 20%, or severe distress symptoms (e.g., continuous lethargy, inability to eat or drink) that required early euthanasia. Randomization of mice to sham/MCAO groups was performed using a random number table by an independent researcher not involved in subsequent experiments. Single-blinding was implemented during data collection and analysis (researchers were unaware of group assignments) to minimize bias. No dropouts occurred during the study, as mice were excluded preoperatively based on predefined criteria (CBF ≥ 70%, intraoperative hemorrhage, or postoperative weight loss >20%) and none met these exclusion criteria after group allocation. Blinding procedures were implemented throughout the study: (1) Randomization and group allocation were performed using a random number table by an independent researcher not involved in subsequent experiments; (2) Data collection (including neurological scoring, tissue sampling, and immunohistochemical/immunofluorescence staining analysis) and statistical analysis were conducted by researchers unaware of the group assignments (single-blinding) to avoid observer bias. Intraoperative efforts to alleviate suffering included continuous monitoring of vital signs (respiration, reflexes) and maintaining rectal temperature at 37 ± 0.5 °C with a heating pad. Postoperatively, analgesia was administered via subcutaneous injection of buprenorphine (0.1 mg/kg) every 12 hours for 48 hours to relieve surgical pain, and mice were observed daily for 72 hours for signs of distress (e.g., reduced mobility, abnormal grooming, weight loss). Mice were sacrificed by cervical dislocation under deep anesthesia (4% isoflurane) to ensure a rapid and painless death, which adheres to the American Veterinary Medical Association (AVMA) Guidelines for the Euthanasia of Animals. After sacrifice, brains were fixed in 4% paraformaldehyde, embedded in paraffin, sectioned (5 μm), deparaffinized, rehydrated, and antigen retrieval was performed.

### 2.5 Immunohistochemistry

Brains were processed for two different staining methods: for immunofluorescence staining, mice were transcardially perfused and brains were cryoprotected in 10%, 20%, and 30% sucrose sequentially before embedding in optimal cutting temperature compound (OCT), and 5 μm cryosections were blocked overnight at 4 °C with 10% goat serum, incubated with anti-CD31 (Abcam, ab9498, 1:100), anti-Ki67 (Abcam, ab15580, 1:200), and anti-15-PGDH (Abcam, ab211745, 1:200), visualized using Alexa Fluor–conjugated secondary antibodies (Invitrogen, Carlsbad, CA, USA, Cat# A-11029, A-11036) and DAPI, and imaged with an Olympus BX63 microscope (Tokyo, Japan); for immunohistochemical staining, brains were fixed in 4% paraformaldehyde, embedded in paraffin, sectioned into 5 μm slices, deparaffinized, rehydrated, and subjected to antigen retrieval, followed by overnight incubation with anti-15-LO antibody (Cayman Chemical, Ann Arbor, MI, USA, Cat# 32800, 1:100), incubation with secondary antibody (ZSGB-Bio, Beijing, China, Cat# ZB-2301, 1:200), visualization with diaminobenzidine, and counterstaining with hematoxylin [10].

### 2.6 Cell transfection

The 15-PGDH siRNA and negative control (NC) siRNA were commercially designed and synthesized by GenePharma (Shanghai, China). The siRNA was designed against the mouse 15-PGDH (Hpgd) transcript. Target site selection was performed by the manufacturer using proprietary algorithms.

Multiple candidate siRNAs targeting different regions of the 15-PGDH transcript were initially evaluated, and the most efficient siRNA was selected for subsequent experiments. A non-targeting scrambled siRNA with no homology to known bovine genes was used as a negative control. Chemically synthesized siRNAs were transiently transfected into cells using standard transfection procedures; no expression vector backbone was involved. Knockdown efficiency was validated at the protein level.

Three shRNA sequences targeting Gpr137b-ps were initially designed and screened. Interference sequences 2 and 3 exhibited no significant knockdown effect on Gpr137b-ps expression; thus, only the most effective interference sequence 1 was utilized in all subsequent in vitro experiments.

### 2.7 Immunofluorescence and immunocytochemistry

For cellular immunostaining (immunocytochemistry), BMVECs were fixed in 4% paraformaldehyde, permeabilized with 0.5% Triton X-100, and blocked with 10% goat serum or 3% BSA. For tissue immunofluorescence, mouse brain cryosections were blocked overnight at 4 °C with 10% goat serum. Primary antibodies against 15-PGDH, CD31, Ki67, and Factor VIII were incubated overnight at 4 °C, followed by Alexa Fluor–conjugated or FITC-conjugated secondary antibodies and DAPI nuclear staining. Images were captured using an Olympus BX63 microscope, and quantitative analysis was performed using ImageJ software.

### 2.8 Tube formation assay

Matrigel (30 μl) was added to the wells of a 96-well plate, and the plate was then incubated at 37 °C for 30 min. BMVECs (5 × 10⁴ cells/ml) were seeded into wells, and the plate was then incubated. Tube formation was visualized using an inverted Nikon microscope and quantified using Image-Pro Plus software. For the rescue experiment involving exogenous 15-PGDH, recombinant human 15-PGDH protein (R&D Systems, Cat# 1260-PG-010) was used. BMVECs transfected with Gpr137b-ps mimics (or negative control mimics) were seeded onto Matrigel-coated wells as described above, and exogenous 15-PGDH was added immediately at a final concentration of 1 μg/mL. The cells were then incubated at 37 °C with 5% CO_2_ for 24 h before tube formation was visualized and quantified using Image-Pro Plus software, consistent with the standard protocol.

### 2.9 Western blotting

Protein extracts made from BMVECs (30–50 μg) were resolved by SDS-PAGE, transferred to membranes (Trans-Blot, Bio-Rad, Hercules, CA), and incubated with antibodies against 15-PGDH, VEGF, cyclin A/D, PCNA, and β-actin. Band intensities of target proteins (15-PGDH, VEGF, cyclin A/D, PCNA) were normalized to β-actin (loading control) using Quantity One Software (Bio-Rad). Relative protein expression was calculated as the ratio of the target protein band intensity to the corresponding β-actin band intensity. As a widely used housekeeping protein, β-actin exhibits consistent expression in BMVECs regardless of experimental treatments (e.g., OGD/R, transfection). Normalization of target protein levels to β-actin eliminates errors caused by uneven sample loading, protein transfer efficiency, or gel staining differences, enabling accurate comparison of protein expression across groups.

### 2.10 qRT-PCR

Total RNA was extracted from cerebral arteries and BMVECs with the use of TRIzol. cDNA was synthesized with a Roche Transcriptor, and quantitative PCR (qPCR) with SYBR Green was performed, and the results were normalized to β-actin. β-actin was chosen as the internal reference gene due to its stable expression in BMVECs under both normoxic and OGD/R conditions. Normalization of target gene (e.g., Gpr137b-ps) expression to β-actin corrects for variations in RNA extraction efficiency, RNA quality, and reverse transcription efficacy, ensuring reliable quantification of relative gene expression levels.

**Table:** 

Gene	Species	Forward primer (5′–3′)	Reverse primer (5′–3′)	Amplicon size (bp)	Accession No.
**15-PGDH (Hpgd)**	Bovine	GCTTCTTCAGGAGACTTCCT	TGAGGTGGTGATGTTGTTGA	124	NM_174059.2
**β-actin (Actb)**	Bovine	CCATGTACGTTGCTATCCAG	GGAGACCAAGAAGGAGGATG	143	NM_173979.3
**GAPDH**	Bovine	GGTGATGCTGGTGCTGAGTA	GAAGGTGGAAGAGTGGGTGT	120	NM_001034034.2

### 2.11 Luciferase reporter assay

Putative binding sites between Gpr137b-ps and the 3’UTR of 15-PGDH were predicted using starBase (version 3.0, http://starbase.sysu.edu.cn/), a tool specialized in RNA-RNA interaction prediction. The computational criteria included complementary base pairing (minimum free energy < −10 kcal/mol) and conservation of binding motifs across species.The pGL3-Basic vector (Promega, Cat# E1751) was used as the backbone for reporter constructs. A fragment of 15-PGDH 3’UTR (containing the two predicted LncRNA-Gpr137b-ps binding sites: Position 762−789 and 1601−1607, as shown in Fig 4B) was amplified by PCR (forward primer: 5’-CTCGAGTTCUAAAAGCAUAUUGGAAGGG-3’; reverse primer: 5’-AGATCTUUCUAAUAACUUAUAACCUUGCC-3’), digested with XhoI/BglII, and cloned into the pGL3-Basic vector to generate the wild-type (WT) construct. Mutant (Mut) constructs (with “ACCUUGCC” mutated to “UGGAAGGG” at both binding sites, matching Fig 4B) were created using the QuikChange Site-Directed Mutagenesis Kit (Agilent, Cat# 200518). All constructs were verified by Sanger sequencing. BMVECs were co-transfected with LncRNA-Gpr137b-ps mimics (or NC mimics), the WT/Mut pGL3 construct, and pRL-TK (internal control) using Lipofectamine 2000. LncRNA-Gpr137b-ps mimics (synthetic oligonucleotides, GenePharma, Shanghai, China) were used to overexpress LncRNA-Gpr137b-ps, with the non-targeting NC oligonucleotide (5’-UUCUCCGAACGUGUCACGUTT-3’) as the control. Luciferase activity was measured 48 h post-transfection via Dual-Luciferase Assay System (Promega).

### 2.12 Fluorescence in situ hybridization (FISH)

RNA probes for Gpr137b-ps, CD31, and β-actin were commercially designed and synthesized by GenePharma (Shanghai, China). Due to proprietary probe design, detailed oligonucleotide sequences are available from the manufacturer upon reasonable request. The probes were labeled with specific fluorophores: Gpr137b-ps with biotin, CD31 with digoxigenin, and β-actin with digoxigenin. Biotin-labeled Gpr137b-ps probes were detected with streptavidin-Alexa Fluor 488 (green, excitation/emission: 495/519 nm, Invitrogen, Cat# S11223, 1:500); digoxigenin-labeled CD31 probes were detected with anti-digoxigenin-Alexa Fluor 594 (red, excitation/emission: 590/617 nm, Invitrogen, Cat# A21366, 1:500); digoxigenin-labeled β-actin probes were detected with anti-digoxigenin-Alexa Fluor 647 (far-red, excitation/emission: 650/668 nm, Invitrogen, Cat# A21469, 1:500). Nuclei were stained with DAPI (blue). Imaging was performed with a Zeiss LSM 880 confocal microscope (Oberkochen, Germany). For quantification, ImageJ software (NIH, USA) was used to analyze the mean fluorescence intensity (MFI) of Gpr137b-ps in CD31-positive endothelial cells (n = 6 independent experiments). The MFI of Gpr137b-ps was normalized to β-actin (internal control) to account for variations in probe hybridization efficiency. The percentage of Gpr137b-ps-positive endothelial cells was calculated as the ratio of cells with Gpr137b-ps MFI > 2-fold the background intensity to total CD31-positive cells.

### 2.13 Cell viability assay

Cell viability post OGD/R was evaluated using the Cell Counting Kit-8 (CCK-8, Dojindo Laboratories, Kumamoto, Japan, Cat# CK04). BMVECs were seeded into 96-well plates at a density of 5 × 10^3^ cells/well and subjected to OGD/R treatment. After reoxygenation for 24 h, 10 μl of CCK-8 reagent was added to each well, and the plates were incubated at 37 °C for 2 h. The absorbance was measured at 450 nm using a microplate reader (Bio-Rad, Hercules, CA, USA). Cell viability was calculated as the percentage of absorbance in treated groups relative to the normoxic control group.

### 2.14 Flow cytometry analysis for cell cycle

BMVECs were seeded in 6-well plates and subjected to OGD/R treatment, siRNA transfection, or Gpr137b-ps mimic transfection as described above. At 24 h after reoxygenation or transfection, cells were harvested by trypsinization (without EDTA), washed twice with cold phosphate-buffered saline (PBS), and fixed in 70% ice-cold ethanol at −20 °C overnight. Fixed cells were centrifuged, resuspended in PBS containing 50 μg/mL propidium iodide (PI, Sigma-Aldrich, St. Louis, MO, USA, Cat# P4170) and 100 μg/mL RNase A (Thermo Fisher Scientific, Waltham, MA, USA, Cat# EN0531), and incubated in the dark at 37 °C for 30 min. Cell cycle distribution was analyzed using a BD FACSCanto II flow cytometer (BD Biosciences, San Jose, CA, USA), and data were processed with ModFit LT 3.0 software (Verity Software House, Topsham, ME, USA) to determine the percentage of cells in G0/G1, S, and G2/M phases.

### 2.15 Statistical analysis

All experiments included both biological and technical replicates: biological replicates refer to independent samples (e.g., different mice or separate BMVEC cultures, with sample sizes specified in the Results), while technical replicates (triplicate readings of the same biological sample) are used to ensure data reliability. All statistical analyses were based on biological replicates to reflect true experimental variation. Results were presented as mean ± SEM. One-way ANOVA was used to compare differences in a single dependent variable (e.g., 15-PGDH protein expression, tube formation length, cell cycle phase distribution) among multiple independent groups (e.g., control, OGD/R, OGD/R + si-15-PGDH, OGD/R + Gpr137b-ps mimic groups), with Dunnett’s post hoc test applied for pairwise comparisons between experimental groups and the control/OGD/R reference group to correct for multiple comparisons. Two-way ANOVA was used to analyze the effects of two independent factors (e.g., “treatment type” [control/OGD/R] and “transfection status” [NC/si-15-PGDH/Gpr137b-ps mimic]) on the dependent variable (e.g., VEGF expression, BMVEC proliferation rate), and Tukey’s post hoc test was utilized for pairwise comparisons between all groups to account for interactions between the two factors. Paired or unpaired t-tests were used for comparisons between two groups as appropriate. Normality of data distribution was verified using the Shapiro-Wilk test prior to ANOVA, and all dependent variables showed a normal distribution (P > 0.05), satisfying the assumption of ANOVA. Homogeneity of variance was confirmed by Levene’s test (P > 0.05), ensuring the validity of the statistical tests. All analyses were performed with GraphPad Prism 5.0 (GraphPad Software, San Diego, CA, USA) and Origin Pro 8.0 (OriginLab, Northampton, MA, USA). Differences were considered statistically significant < 0.05.

### 2.16 Ethics approval

The current research obtained approval from the Institutional Animal Care and Use Committee (IACUC) of Tsinghua University College of Medicine and adhered to the principles outlined in the Declaration of Helsinki and relevant institutional guidelines. For recombinant DNA techniques involved in the study, approval was obtained from the Biological Safety Committee of Tsinghua University, with strict adherence to safety regulations throughout the study. The registration procedure complied with the guidelines of the International Committee of Medical Journal Editors (ICMJE).

## 3 Results

### 3.1 15-PGDH expression rises in BMVECs after stroke

**Fig 1:**
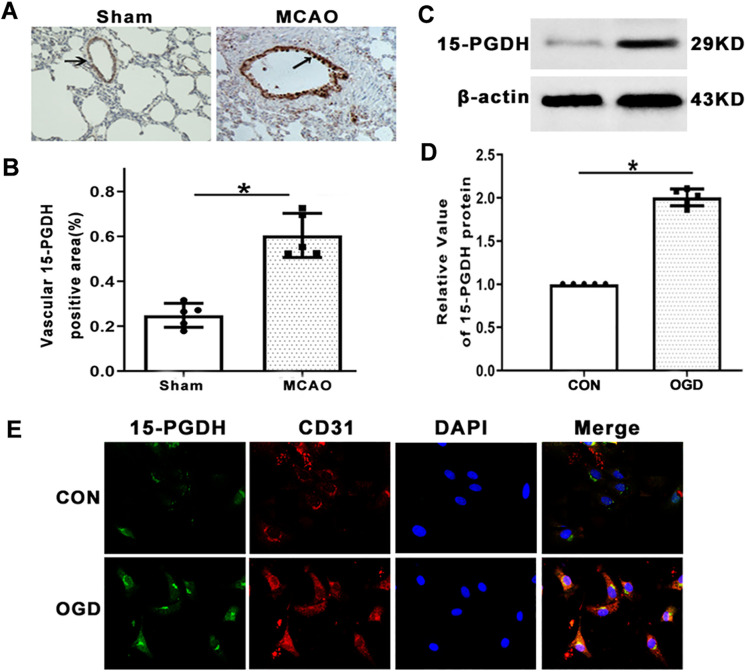
15-hydroxyprostaglandin dehydrogenase (15-PGDH) expression is increased in the brain artery endothelium of post-stroke mice and OGD/R-induced BMVECs. (A) Immunohistochemical staining of 15-PGDH in brain arteries from sham-control and MCAO mice. Immunoreactive 15-PGDH was mainly expressed in brain vascular intima (n = 10, * *P* < 0.05). (B) Quantitative analyses of 15-PGDH-positive immunostaining per vascular area (adventitia + media + intima + lumen). (C) The protein expression of 15-PGDH in ICAs from sham-control and middle cerebral artery occlusion (MCAO) mice was detected by Western blot. (D) The quantitative data for 15-PGDH proteins. (n = 6, * *P* < 0.05). (E) The location of 15-PGDH was confirmed with immunofluorescence staining. Brain microvascular endothelial cells (BMVECs) were fixed and stained with anti-15-PGDH (green), anti-CD31 (red), and DAPI to stain nuclei (blue). Merged images show 15-PGDH colocalizes to CD31 (a marker of ECs). Scale bars: 25μm (magnification: 400× for A-B, 200× for E). The images shown are representative of at least three independent experiments. All values are represented as the mean±S.E.M.

Immunohistochemical analysis indicated an increased 15-PGDH expression post-MCAO, with expression predominantly localized to the vascular intima (Fig 1A, 1B). This endothelial localization was confirmed by co-staining with CD31 (an endothelial cell-specific marker), verifying that 15-PGDH expression was derived from endothelial cells rather than mural cells. Compared to controls, the vascular wall in the MCAO group showed apparent thickening, which was not a staining artifact as evidenced by consistent morphological observations across multiple sections and preserved vascular structure. Due to the lack of quantitative measurements, the nature of this thickening (e.g., hypertrophy or edema) remains to be further clarified.

### 3.2 Expression and localization of 15-PGDH in ischemic BMVECs

Western blotting showed significantly higher 15-PGDH protein levels in BMVECs exposed to OGD/R conditions compared to normoxic controls (n = 5 independent experiments, P < 0.05; Fig 1C, 1D). Immunofluorescence staining confirmed that 15-PGDH colocalized with the endothelial marker CD31 (n = 5 independent experiments; Fig 1E), and revealed apparent nuclear localization of 15-PGDH under hypoxic conditions based on visual observation (quantitative analysis of nuclear/cytoplasmic distribution was not performed). Notably, the timing of 15-PGDH elevation post-OGD (early, late, or biphasic) was not determined in this study.

### 3.3 15-PGDH and endothelial cell migration and proliferation post-OGD/R

**Fig 2:**
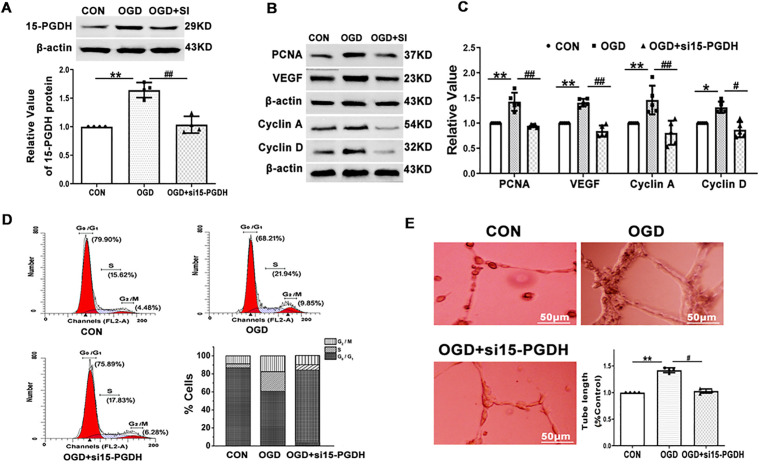
15-PGDH mediates BMVECs’ proliferation, migration, and cell-cycle progression after OGD/R. (A) 15-PGDH protein expression was down-regulated after transfecting the 15-PGDH siRNA sequence into BMVECs. **(B) (C)** Analysis of PCNA (proliferating cell nuclear antigen), VEGF (vascular endothelial growth factor), cyclin A, and cyclin D expression in BMVECs after OGD/R with silencing of 15-PGDH by Western blotting (n = 6, * *P* < 0.05, ^#^ *P* < 0.05, ** *P* < 0.01, ^##^ *P* < 0.01). **(D)** The number of cells in each phase of the cell cycle was examined by fluorescence-activated cell sorting. The bar graph shows the number of cells in each phase of the cell cycle. Cells accumulated in S and G2/M under OGD/R. This was reversed by 15-PGDH inhibition (n = 6). **(E)** BMVECs were subjected to the tube formulation assay. 15-PGDH siRNA inhibited cell tube formation induced by OGD/R (n = 6, ** *P* < 0.01, ^#^ *P* < 0.05). All values are presented as the mean±SEM.

Western blots confirmed successful 15-PGDH knockdown (n = 6 independent experiments, *P* < 0.01; Fig 2A), and this silencing significantly reduced OGD/R-mediated expression of PCNA and VEGF compared to cells transfected with negative control (NC) siRNA (knockdown control, *P* < 0.05 for both; Fig 2B, 2C). In this study, 15-PGDH silencing significantly inhibited OGD/R-induced tube formation in BMVECs (Fig 2E), consistent with the reduced proliferation and migration of these cells.

### 3.4 15-PGDH promotes cell cycle progression of endothelial cells

Cyclin A and cyclin D expression was increased when exposed to OGD/R conditions; however, they were reduced in BMVECs treated with 15-PGDH shRNA (Fig 2B, 2C). Flow cytometry was then performed to assess the rate of cell cycle progression, and it was discovered that a greater number of cells were in the S and G2/M phases than in controls. Conversely, 15-PGDH knockdown reduced the number of cells in the S and G2/M phases while showing a greater % of cells in the G0/G1 phase versus controls (Fig 2D). Notably, while 15-PGDH is canonically a prostaglandin-degrading enzyme, this pro-proliferative role in BMVECs may reflect cell-type specificity or adaptation to the ischemic microenvironment—where prostaglandin turnover is remodeled to prioritize vascular repair, consistent with previous reports of context-dependent functions of 15-PGDH in endothelial cells [10,11].

### 3.5 Endothelial cells express Gpr137b-ps after stroke

**Fig 3:**
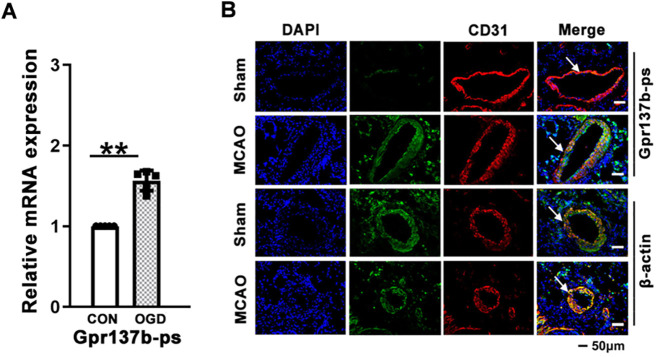
Gpr137b-ps is upregulated in the brain and BMVECs of MCAO mice. **(A)** The up-regulation of relative expression of Gpr137b-ps caused by OGD/R (n = 6, ** *P* < 0.01). (**B)** Fluorescence in situ hybridization was used to determine the distribution of Gpr137b-ps in mice cerebral tissues of sham group and MCAO group, scale bar = 50 μm (magnification: 200×). Merged images show Gpr137b-ps co-localizes to brain vascular endothelial cells. All values are presented as the mean±SEM.

qRT-PCR results showed increased Gpr137b-ps expression in BMVECs subjected to OGD/R conditions when compared to controls (Fig 3A). Fluorescence in situ hybridization results showed that Gpr137b-ps expression was upregulated and was also present in the nucleus in vitro and in vivo,and was colocalized with CD31 (Fig 3B). FISH also confirmed colocalization of Gpr137b-ps with CD31 (endothelial marker), verifying its endothelial-specific expression. Additionally, Pearson correlation analysis showed a negative correlation between Gpr137b-ps and 15-PGDH expression at the tissue level. Notably, while Gpr137b-ps is upregulated under ischemic conditions (where angiogenesis is beneficial), subsequent results demonstrated its anti-angiogenic role—a functional tension that will be explored in detail in later sections.

### 3.6 Gpr137b-ps acts as a negative regulator of 15-PGDH

**Fig 4:**
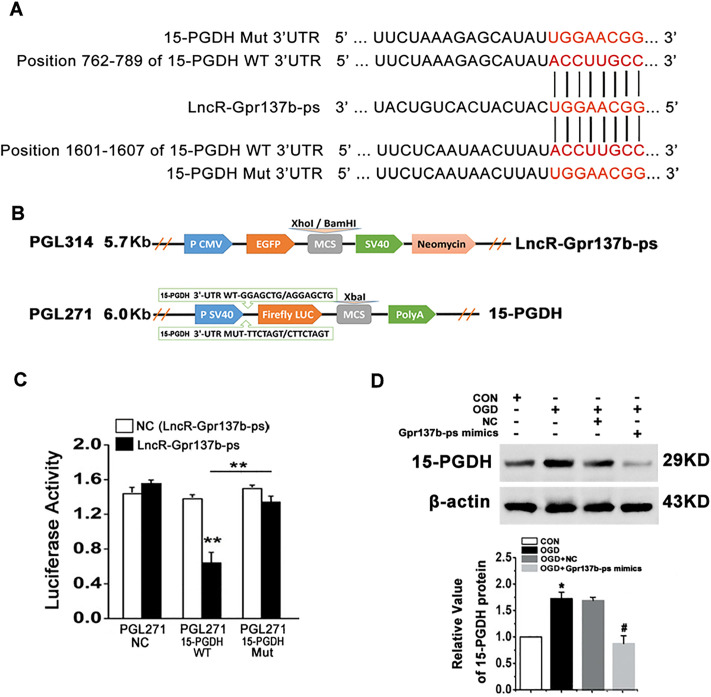
Gpr137b-ps binds to the 15-PGDH promoter and regulates 15-PGDH expression. (A) The binding sites between Gpr137b-ps and the 15-PGDH gene predicted by the starBase website. (**B)** Dual luciferase vector and mutant plasmids were designed based on binding sites with the highest 15-PGDH and Gpr137b-ps scores. (**C)** Dual luciferase assays were used to validate the interactions between 15-PGDH and Gpr137b-ps (n = 3, ** *P* < 0.01). (**D)** The protein levels of 15-PGDH in cells exposed to control, OGD, OGD + non-targeting Gpr137b-ps shRNA, and OGD + shGpr137b-ps, the Gpr137b-ps silencing reduced the levels of 15-PGDH under OGD (n = 5, * *P* < 0.05, ^#^ *P* < 0.05). PGL271 indicates plasmid vector; mut, mutant type; NC, negative control; CON, control; and WT, wild type. All values are presented as the mean±SEM (Fig 4).

Bioinformatics analysis via starBase (version 3.0), a tool specialized in RNA-RNA interaction prediction, identified two putative binding sites (positions 762−789 and 1601−1607) in the 15-PGDH transcript for Gpr137b-ps, based on criteria including complementary base pairing, minimum free energy < −10 kcal/mol, and conservation of binding motifs (Fig 4A). To explore the potential regulatory relationship between Gpr137b-ps and 15-PGDH, dual-luciferase assays were performed, showing that Gpr137b-ps overexpression reduced the activity of wild-type 15-PGDH 3’UTR reporter constructs but not mutant constructs (Fig 4B, 4C)—supporting that the predicted binding sites are required for this regulatory effect. Additionally, transient transfection of Gpr137b-ps mimics in BMVECs reduced 15-PGDH protein levels (Fig 4D). Notably, direct binding between endogenous Gpr137b-ps and 15-PGDH mRNA (e.g., via RNA pull-down or RIP assays) and effects on 15-PGDH mRNA stability were not evaluated in this study, so the regulatory mechanism remains to be fully elucidated.

While our data demonstrate that Gpr137b-ps suppresses 15-PGDH protein levels and binds to its 3’UTR (Fig 4), the specific molecular mechanism by which Gpr137b-ps reduces 15-PGDH remains unclear. We acknowledge that we have not addressed whether this regulation occurs via RNA degradation, transcriptional repression, ceRNA competition, or other post-transcriptional pathways. Based on the dual-luciferase assay results—showing reduced activity of the wild-type 15-PGDH 3’UTR reporter (but not the mutant) upon Gpr137b-ps overexpression—we speculate that Gpr137b-ps may regulate 15-PGDH at the post-transcriptional level, potentially via inhibiting translation or modulating mRNA stability. However, these hypotheses require further validation with additional experiments (e.g., RNA stability assays, RNA pull-down, or RIP) to confirm direct binding and clarify the downstream regulatory cascade. The current study focuses on establishing the functional relationship between Gpr137b-ps and 15-PGDH in angiogenesis, and future work will prioritize elucidating the exact molecular mechanism underlying this regulation.

### 3.7 Gpr137b-ps effect on endothelial proliferation and angiogenesis is dependent on 15-PGDH

**Fig 5:**
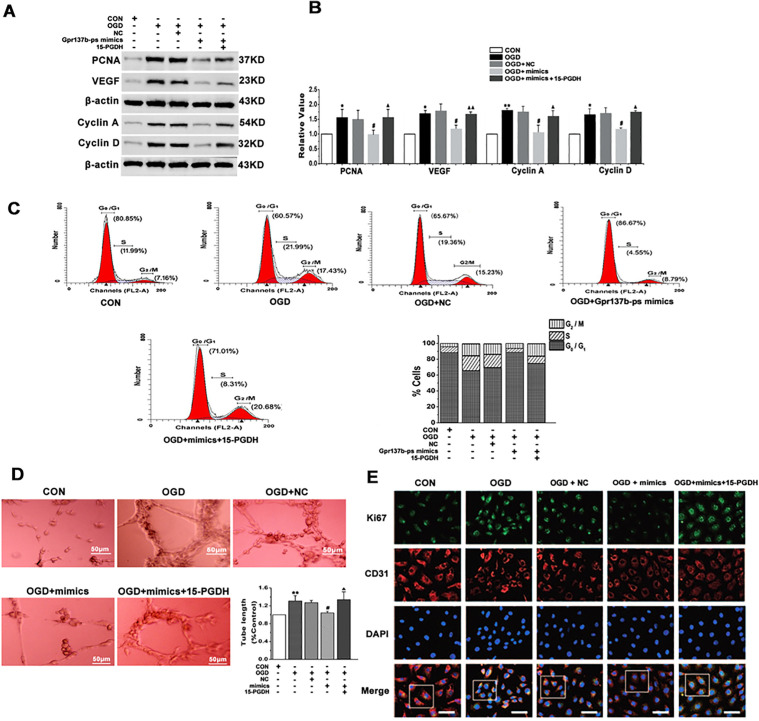
Gpr137b-ps is involved in 15-PGDH-induced brain microvascular endotheliogenesis after ischemic stroke. **(A)** Cells were transduced with lentiviral vectors expressing shRNA targeting Gpr137b-ps. Interference sequence 1 was selected for subsequent experiments based on knockdown efficiency (** *P* < 0.01, vs. Non-targeting Gpr137b-ps shRNA). (**B)** Detection of PCNA, VEGF, cyclin A, and cyclin D protein expression by Western blotting (n = 6, * *P* < 0.05 OGD vs control, # *P* < 0.05 OGD + shGpr137b-ps vs OGD + non-targeting Gpr137b-ps shRNA, ^△^ *P* < 0.001 OGD + shGpr137b-ps + 15-PGDH vs. OGD + shGpr137b-ps). (**C)** The number of cells in each phase of the cell cycle was examined by fluorescence-activated cell sorting. The bar graph shows the number of cells in each phase of the cell cycle (n = 6). (**D)** BMVECs were subjected to the tube formulation assay. Knockdown of Gpr137b-ps inhibited cell tube formation induced by OGD, which was attenuated by exogenous 15-PGDH (1μmol/mL) with 24-hour incubation. **(E)** Ki67, a marker of cell proliferation, was used to evaluate the proliferative capacity of BMVECs. Double immunofluorescence staining for anti-Ki67 (green), anti-CD31 (red), and DAPI to stain nuclei (blue) in BMVECs with the indicated treatment. Merged images show Ki67 co-localizes to brain vascular endothelial cells. Co-localization was most apparent after OGD; these effects were blocked by knockdown of Gpr137b-ps and activated by 15-PGDH. Scale bars are 10μm. The images shown are representative of at least three independent experiments.

BMVECs overexpressing Gpr137b-ps showed significant reductions in PCNA, VEGF, cyclin A, and cyclin D proteins compared to controls (Fig 5B). Flow cytometry showed increases in G0/G1 phase arrest and decreases in S phase entry in cells that overexpressed Gpr137b-ps (Fig 5C). Flow cytometry showed significant G0/G1 phase arrest in cells overexpressing Gpr137b-ps. This arrest indicates that Gpr137b-ps inhibits BMVEC proliferation by blocking cell cycle progression from G0/G1 to S phase, which aligns with reduced cyclin A/D expression (Fig 5B) and impaired angiogenic ability (Fig 5D). Tube formation assays confirmed that cells overexpressing Gpr137b-ps had reduced angiogenic ability, as indicated by decreased total tube length, while the addition of exogenous recombinant 15-PGDH (1 μmol/mL, R&D Systems) immediately after seeding onto Matrigel restored their ability to form tubes (Fig 5D). BMVECs treated with Gpr137b-ps mimics showed a reduction in Ki67 staining in Ki67 and CD31 immunostaining (Fig 5E).

## 4 Discussion

Our results demonstrate that ischemia upregulates 15-PGDH in vascular walls of cerebral arteries, primarily surrounding the vascular intima. This finding was consistent with previous studies by our group, which showed that 15-PGDH has a protective role in smooth muscle cells cultured under OGD/R conditions [8,10,11]. In the present work, we have shown that endothelial 15-PGDH itself exerts a pro-angiogenic role by regulating BMVEC proliferation, migration, and cell-cycle progression (with concurrent changes in VEGF expression). However, the Gpr137b-ps/15-PGDH axis forms a feedback regulatory loop: both molecules are upregulated by ischemia, but Gpr137b-ps (a dedicated negative regulator) suppresses 15-PGDH expression to limit excessive angiogenic responses, ensuring balanced vascular repair—consistent with our gain- and loss-of-function data. Our findings are also consistent with older work describing the proximate mapping of prostaglandin pathway enzymes, as agents in vascular remodeling [12–15], and recently supplemented by work describing hypoxia-driven angiogenic programs [16–18].

Notably, our finding that 15-PGDH knockdown reduces angiogenesis in BMVECs appears contradictory to classic prostaglandin biology, where 15-PGDH-mediated degradation of pro-angiogenic PGE_2_ would be expected to inhibit angiogenesis. This discrepancy likely stems from the context-dependent function of 15-PGDH, which varies by cell type and microenvironment [10,11]. In BMVECs under ischemic conditions, 15-PGDH may exert pro-angiogenic effects via non-canonical pathways independent of PGE_2_ degradation—for example, by modulating PI3K/Akt or cAMP/PKA signaling (as highlighted in the Introduction) that directly regulate endothelial proliferation and migration [11,26]. Consistent with this, previous studies have reported pro-angiogenic roles of 15-PGDH in pulmonary artery endothelial cells under hypoxia [11], supporting that its function is not restricted to prostaglandin degradation. Additionally, the ischemic microenvironment may remodel prostaglandin metabolism, with other prostaglandin species (e.g., PGF_2_α) or downstream mediators overriding the classic PGE_2_-dependent pathway [2,12]. Thus, 15-PGDH’s pro-angiogenic role in BMVECs represents a cell-type and context-specific adaptation to ischemia, rather than a contradiction to known biology.

We acknowledge that our finding of 15-PGDH’s pro-angiogenic role in BMVECs appears to conflict with established evidence of its anti-proliferative, anti-angiogenic functions in other systems (e.g., cancer, inflammatory diseases) and recent work showing that 15-PGDH inhibition is neuroprotective after stroke. This apparent discrepancy arises from three key factors: first, cell type specificity,—15-PGDH exerts distinct effects in endothelial cells versus neurons, glial cells, or cancer cells. While it may suppress proliferation in cancer cells via PGE_2_ degradation [10,15], our data show it promotes BMVEC proliferation and angiogenesis, consistent with reports of its pro-angiogenic role in hypoxic pulmonary artery endothelial cells [11]. Second, pathological stage dependency—ischemic stroke involves sequential phases: early acute injury (characterized by ferroptosis, oxidative stress) and later vascular repair (requiring angiogenesis). The study focuses on early neuroprotection by inhibiting 15-PGDH to reduce neuronal ferroptosis, whereas our work targets the vascular repair phase, where BMVEC-derived 15-PGDH supports angiogenesis. Third, functional pleiotropy—15-PGDH modulates multiple pathways (prostaglandin metabolism, PI3K/Akt, ferroptosis) that are activated in a context-dependent manner. Thus, 15-PGDH inhibition may be beneficial for neuroprotection in the acute phase, while its upregulation in BMVECs facilitates vascular repair in the subacute phase—these are complementary rather than contradictory findings, reflecting the complex, cell-specific roles of 15-PGDH in ischemic stroke.

More specifically, rapidly emerging studies highlight HIF signaling and endothelial metabolic reprogramming in ischemic angiogenesis [19], and our data suggest 15-PGDH supports BMVEC proliferation as an indirect mediator of VEGF-dependent cell-cycle progression. Critically, our data suggest that this pro-angiogenic role of 15-PGDH is likely regulated by Gpr137b-ps: Gpr137b-ps suppresses 15-PGDH, which correlates with reduced angiogenic activity, supporting an overall anti-angiogenic role of the Gpr137b-ps/15-PGDH axis—consistent with our conclusion that this axis may modulate post-stroke angiogenesis via inhibiting endothelial function, based on correlative and in vitro evidence. This supports a functional role for pseudogenes like Gpr137b-ps in endothelial cells: Gpr137b-ps is upregulated under OGD/R and MCAO, suppresses 15-PGDH protein levels, and reduces angiogenic outcomes—advancing our exploration of how pseudogenes and lncRNAs act as competitive endogenous RNAs (ceRNAs) to regulate angiogenesis and vascular repair [20–23]. Notably, Gpr137b-ps-induced G0/G1 phase arrest directly contributes to its anti-angiogenic effect: by blocking BMVEC progression through the cell cycle, Gpr137b-ps reduces the pool of proliferative endothelial cells capable of migration and tube formation. This is consistent with cyclin A/D downregulation (key regulators of G1 → S transition) in Gpr137b-ps-overexpressing cells, reinforcing that the Gpr137b-ps/15-PGDH axis inhibits angiogenesis via dual mechanisms: suppressing 15-PGDH and inducing cell cycle arrest. This also provided a critical framework, which, focusing on ischemic brain angiogenesis, we examined Gpr137b-ps potentially as a factor, and ultimately brought pseudogene work into neurovascular repair, leap-frogging from previous investigations examining pseudogenes with atherosclerosis and cancer pathology [24].

Furthermore, our findings are reinforced by another recent conclusion that emphasizes hypoxia-associated cyclin control modulating the underlying endothelial proliferation under ischemia [23–25], our cell-cycle assessments position 15-PGDH as capable of signaling, accelerating the transition from G1 to S/M, while 15-PGDH knockdown cells are in G0/G1. This would echo recent single-cell transcriptomic studies describing phase-specific reprogramming of endothelial cells during ischemic sprouting angiogenesis [26].

Recent advances in translational research have shown that post-stroke angiogenesis is relevant to functional recovery in patients, while circulating angiogenic biomarkers are predictive of functional rehabilitation in stroke patients [27]. Our findings suggested that the Gpr137b-ps/15-PGDH axis is a putative mechanistic regulator, with preliminary correlative and in vitro evidence supporting its potential as a modulator to promote neurovascular repair—though further in vivo validation is needed.

Notably, while Gpr137b-ps and 15-PGDH are naturally upregulated post-stroke as a tissue adaptive response, our findings reveal that their dysregulated balance (excessive Gpr137b-ps leading to 15-PGDH suppression) limits optimal angiogenesis. To actively mitigate ischemic damage beyond the passive tissue response, we propose inducible strategies targeting this axis: first, inducible inhibition of Gpr137b-ps (e.g., via tissue-specific siRNA or antisense oligonucleotides [ASOs] activated by stroke-related hypoxia or inflammation) to relieve its repression on 15-PGDH, thereby enhancing endothelial proliferation and vascular repair. Second, conditional activation of 15-PGDH (e.g., via hypoxia-responsive promoters driving 15-PGDH overexpression) to amplify its pro-angiogenic effects specifically in ischemic brain regions, avoiding off-target impacts. Third, small-molecule modulators that disrupt the Gpr137b-ps/15-PGDH interaction—inducible by oral administration post-stroke—could directly restore 15-PGDH activity. These strategies are distinct from the natural tissue response: they actively correct the pathological imbalance of the Gpr137b-ps/15-PGDH axis, rather than relying on intrinsic adaptive mechanisms. Preclinical studies have validated similar inducible approaches for lncRNA-targeted therapy in stroke [20], and CRISPR-based conditional activation of pro-angiogenic genes has shown promise in vascular repair, supporting the feasibility of our proposed strategies. Future work will focus on developing hypoxia-inducible vectors or small molecules to test these interventions, with the goal of translating the Gpr137b-ps/15-PGDH axis into a clinically actionable target for ischemic stroke.

Compared to previous work specifically focused on pseudogenes (13,24), our work is the first to demonstrate directly that Gpr137b-ps regulates brain ischemic angiogenesis, reiterating the unique and impactful component of stroke recovery that our finding denotes.

Limitations: This study was a single-center study with a model of experimental stroke; we did not center our discussion on a more advanced rationale for mechanistic validation of genome editing (i.e., RNA pull-down, co-IP, in vivo knock-ins), and we also did not utilize any patient-based clinical samples. Additionally, while our data show that 15-PGDH modulation is accompanied by changes in VEGF expression, we have not performed pharmacological or genetic inhibition of VEGF to directly confirm it as a downstream effector of 15-PGDH in angiogenesis. Future studies will address this by investigating whether VEGF inhibition abrogates the angiogenic effects of 15-PGDH, to clarify their functional hierarchy. Future studies would likely employ applications of CRISPR-based genomic editing of pseudogenes and high-throughput sequencing as modalities to ultimately validate the therapeutic relevance of the Gpr137b-ps/15-PGDH axis in clinical stroke populations [27]. Another limitation is that while FISH and luciferase reporter assays support an interaction between Gpr137b-ps and 15-PGDH, we have not performed RNA pull-down or RNA immunoprecipitation (RIP) assays to confirm their direct physical binding. This means the current mechanistic link relies on indirect evidence, and future studies will employ these techniques to validate the direct interaction.Furthermore, the observed regulatory effects of Gpr137b-ps on 15-PGDH and angiogenesis may be indirect, as other regulatory RNAs (e.g., microRNAs, other lncRNAs) or intermediate signaling molecules not explored in this study could also be involved, which warrants further investigation. Additionally, this study has several other limitations worth noting. First, the MCAO model primarily focuses on arterial tissue analysis, which may not fully represent the biological characteristics of peri-infarct microvessels—the key site of post-stroke angiogenesis. Second, while the in vitro OGD/R model effectively mimics ischemic/hypoxic stress, it cannot fully recapitulate the complex in vivo microenvironment (e.g., interactions with immune cells, extracellular matrix components) that regulates endothelial behavior after stroke, potentially limiting the translational relevance of in vitro findings. Third, we did not perform in vivo gain- and loss-of-function experiments for Gpr137b-ps or 15-PGDH, which prevents direct validation of the axis’s regulatory role in post-stroke angiogenesis in living organisms. Future studies will address these gaps by focusing on peri-infarct microvessel samples, optimizing in vitro models to better mimic in vivo conditions, and conducting in vivo genetic manipulation experiments (e.g., conditional knockout or overexpression) to confirm the functional significance of the Gpr137b-ps/15-PGDH axis.

Overall, we conclude that the Gpr137b-ps/15-PGDH axis is a putative new modulator of angiogenesis with potential functional relevance to brain recovery after ischemic stroke, where it is associated with endothelial proliferation, migration, and cell-cycle transition—based on correlative data and in vitro experiments, rather than definitive mechanistic proof. Our gain- and loss-of-function data support an association and partial dependency of this axis on post-stroke angiogenesis (rather than a definitive causal mechanism), highlighting it as a new tractable target for vascular repair in stroke recovery from an experimental research perspective.

## 5 Conclusion

This work provides consistent evidence that the Gpr137b-ps/15-PGDH axis negatively regulates post-stroke angiogenesis, as demonstrated by our gain- and loss-of-function assays: Gpr137b-ps (an angiogenesis inhibitor) suppresses 15-PGDH (a pro-angiogenic factor), thereby reducing endothelial proliferation, migration, and VEGF-driven cell-cycle progression. This axis function resolves the previous wording inconsistency, with data clearly showing Gpr137b-ps itself inhibits angiogenesis by repressing 15-PGDH. Given the non-physiological dose of exogenous 15-PGDH used in the rescue experiment, the conclusion of ‘partial dependency on 15-PGDH’ is supported by the available data but should be interpreted with caution, as it may not reflect in vivo physiological conditions. This is the first evidence that the pseudogene Gpr137b-ps regulates vascular repair in the context of ischemic stroke, contributing to the understanding of pseudogene biology in the context of stroke recovery. While the study is limited in experimental design with no use of patient samples, the pathway we have identified provides preliminary preclinical evidence for a potential therapeutic target, though its clinical application to induce vascular repair and functional recovery following ischemic stroke requires further validation.

## Abbreviations

AA: arachidonic acid
BMVECs: brain microvascular endothelial cells
Gpr137b-ps: G protein-coupled receptor 137 b-pseudogene
MCAO: middle cerebral artery occlusion
OGD: oxygen-glucose deprivation/reoxygenation
PCNA: proliferating cell nuclear antigen
PGs: prostaglandins
QRT-PCR: quantitative real-time polymerase chain reaction
VEGF: vascular endothelial growth factor
15-LO: 15-lipoxygenase
15-PGDH: 15-hydroxyprostaglandin dehydrogenase
